# Rare Presentation of Splenic Abscess Secondary to Clostridioides difficile Infection in a Patient With Systemic Sclerosis: A Case Report

**DOI:** 10.7759/cureus.76754

**Published:** 2025-01-01

**Authors:** Matthew J Van Ligten, Akhila Nalla, Sara Shihab, Wayne A Martini

**Affiliations:** 1 Emergency Medicine, Mayo Clinic Alix School of Medicine, Phoenix, USA; 2 Women's Health Internal Medicine, Mayo Clinic, Phoenix, USA; 3 Emergency Medicine, Mayo Clinic, Phoenix, USA

**Keywords:** case report, clostridioides difficile, rare infection, splenic abscess, systemic sclerosis

## Abstract

Splenic abscesses are rare but serious infections often linked to immunosuppressive conditions. While *Clostridioides difficile* is well-known for causing colitis, its occurrence in locations outside the gastrointestinal tract, like the spleen, is exceedingly rare. This report highlights a unique case of a *C. difficile* splenic abscess in a patient with systemic sclerosis and multiple comorbidities.

A 73-year-old female with a history of systemic sclerosis, recent colectomy with end ileostomy for fulminant *C. difficile* colitis, and other significant comorbidities, presented with abdominal pain, nausea, and decreased ostomy output. A CT scan revealed an organized splenic infarction with surrounding fluid collection, as well as a right parastomal hernia. The initial management included intravenous (IV) piperacillin-tazobactam, oral vancomycin, and IV fluids. Surgical consultation determined that immediate intervention was unnecessary, and a follow-up was planned. An interventional radiologist performed aspiration of the fluid collection, which was positive for *C. difficile*. Infectious Disease specialists subsequently recommended a 3-week course of oral metronidazole, and the patient was discharged with symptom control on an oral pain regimen.

There are many diagnostic and therapeutic challenges posed by extraintestinal *C. difficile* infections. Splenic abscesses caused by *C. difficile* are rare, and their variable clinical presentations can lead to delays in diagnosis. Currently, no standardized guidelines exist for managing such infections, making individualized treatment essential. The successful management of our patient’s infection involved advanced imaging, percutaneous drainage, and tailored antibiotic therapy. Given the potential for antibiotic resistance and recurrence, prolonged follow-up and careful management are recommended.

Extraintestinal *C. difficile *infections, such as splenic abscesses, are rare and complex, highlighting the need for further research to develop standardized diagnostic and therapeutic protocols. This case contributes to the limited literature on this rare entity, underscoring the importance of a multidisciplinary approach in optimizing patient outcomes.

## Introduction

Splenic abscesses, though rare, represent a significant medical challenge with potentially severe outcomes [1]. They are typically attributed to hematogenous spread, superinfection of hematomas, or contiguous spread from neighboring abdominal infections [1]. This condition is estimated to appear in only 0.05% to 0.7% of autopsies, often associated with specific comorbidities such as diabetes mellitus, pancreatic diseases, and immunosuppressive conditions [1,2]. The bacterial spectrum implicated in splenic abscesses is diverse, with common isolates, including *Escherichia coli*, Enterococcal species, and anaerobes, often presenting as polymicrobial infections in up to one-third of the cases [1]. 

While *Clostridioides difficile* is predominantly recognized as a cause of colitis, extraintestinal manifestations like splenic abscesses are exceedingly rare, with fewer than ten cases reported in the literature [2-8]. Risk factors for such infections include recent gastrointestinal surgeries, previous *C. difficile *infections, hospitalization, and antibiotic exposure [2]. Given the rarity of C. difficile in the spleen, the optimal treatment protocol remains unclear, and management typically involves prolonged antimicrobial therapy and percutaneous drainage [2]. We present a case of a 73-year-old female with a splenic abscess with an atypical pathogen. 

## Case presentation

The patient is a 73-year-old female with a significant medical history, including interstitial lung disease, immunodeficiency secondary to rituximab, methotrexate, and prednisone, systemic sclerosis, rheumatoid arthritis, interstitial lung disease, and hypertension, arriving in her personal vehicle from a skilled nursing facility. She previously presented to the hospital with a chief complaint of abdominal pain and diarrhea. During her hospitalization, she required an ICU stay for hypotension and underwent a colectomy due to community-acquired fulminant *Clostridioides difficile *colitis. She was treated with IV metronidazole 500 mg for ten days as well as a vancomycin enema. She was not given oral vancomycin since source control was felt to be effective with colectomy. She was discharged with a total of three weeks of oral metronidazole 500 mg and planned follow-up at the Infectious Disease department. 

Ninety-one days later, she presented to the emergency department with acute-on-chronic worsening abdominal pain, nausea, and reduced ostomy output. Two days prior to arrival, she discontinued oral hydromorphone for pain management as it was no longer effective. She also reported associated symptoms of chills, night sweats, and weight loss without a recorded fever at home.

**Figure 1:**
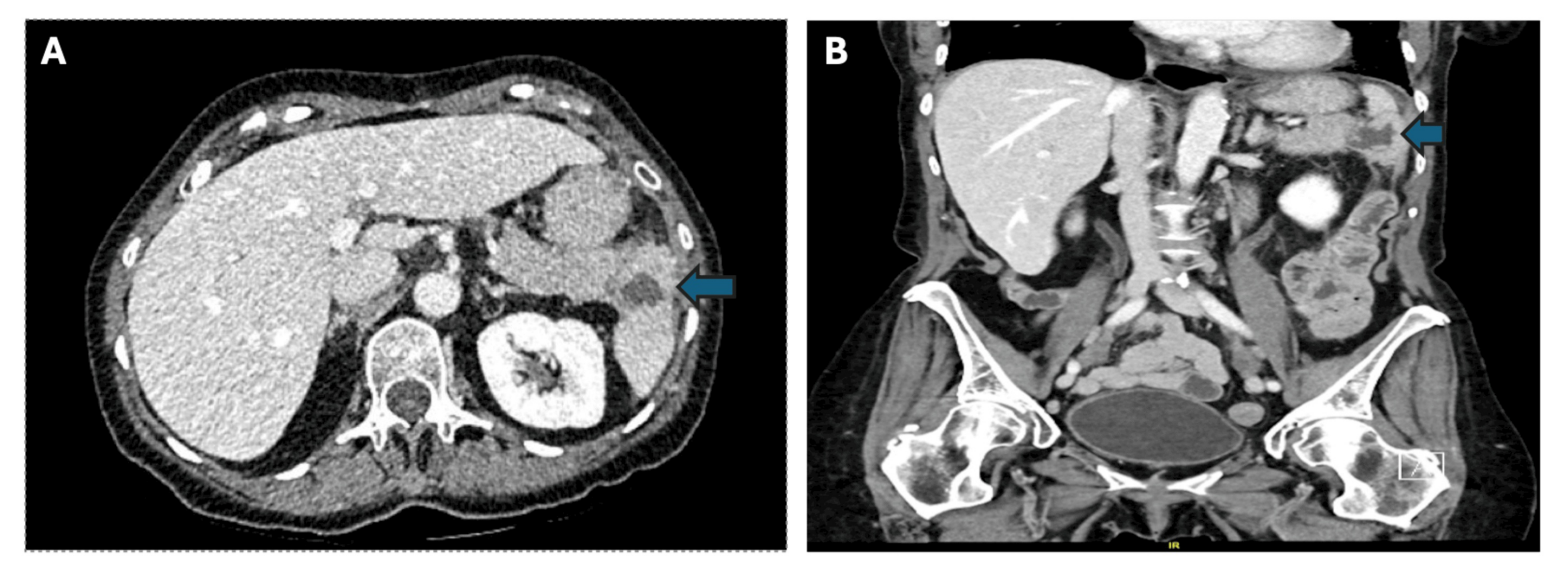
CT of the abdomen and pelvis CT of the abdomen and pelvis with contrast showing initial splenic fluid collection in the axial (A) and coronal (B)views. Fluid collection is denoted by blue arrows

Upon arrival at the emergency department, the patient was hemodynamically stable and afebrile. Physical examination revealed fullness and tenderness around the stoma site with concomitant left upper quadrant abdominal tenderness. Laboratory tests demonstrated leukocytosis (14.9) with neutrophilic predominance, while electrolytes and lactate levels were within normal limits. Given the reduced ostomy output and leukocytosis, a consultation with General Surgery was requested for further evaluation and management. A CT scan of the abdomen and pelvis showed a previously noted splenic infarction, now complicated by an organized fluid collection (Figure 1). The patient was started on oral vancomycin and intravenous piperacillin-tazobactam for broad-spectrum antibiotic coverage and was admitted under Internal Medicine for further evaluation and work-up.

**Figure 2:**
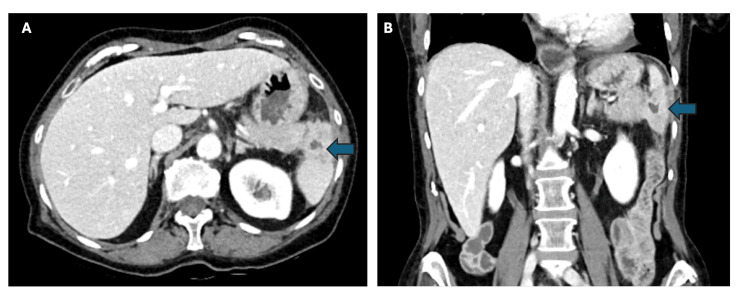
CT of the abdomen and pelvis with contrast CT of the abdomen and pelvis with contrast showing splenic fluid collection in the axial (A) and coronal (B) views status post drainage and antibiotics. Fluid collection is denoted by blue arrows​.

During her hospitalization, the patient underwent radiology-guided aspiration of the fluid collection, with 1-2 mL of thick pale yellow material which cultured *C. difficile*. Blood and urine cultures did not result in any growth. The Infectious Disease team recommended discontinuing IV piperacillin-tazobactam while continuing oral metronidazole 500 mg three times daily and oral vancomycin 125 mg four times daily for a total of three weeks. Approximately two weeks later, she returned to the emergency department with worsening leukocytosis and fatigue. A repeat CT scan revealed evidence of an improving infection, and she was advised to follow up with the outpatient Infectious Disease clinic (Figure 2). An ultrasound performed 6 weeks after initial abscess aspiration showed continued evolution of splenic abscess with scant fluid remaining. She was ultimately kept on metronidazole 500 mg every 8 hours for a total of 8 weeks, and vancomycin 125 mg four times daily for four weeks with resolution of her symptoms. She elected to avoid any further immune-compromising medications after conversations about the risks versus benefits with the Rheumatologist. 

## Discussion

There are many unique and complex challenges associated with extraintestinal *Clostridioides difficile* (*C. difficile*) infections, particularly in the context of splenic abscesses. The rarity of such infections complicates both diagnosis and management, as the available literature primarily consists of case reports and small case series, which are valuable but inherently limited in scope [2,9,10]. This case highlights the need for a better understanding of extraintestinal *C. difficile* infections to establish clearer diagnostic and treatment protocols.

Diagnosing splenic abscesses, especially those caused by *C. difficile*, presents a unique set of challenges. The clinical presentation can vary significantly, often mimicking other conditions and leading to diagnostic delays [1,2]. Laboratory markers, such as liver function tests and C-reactive protein levels, may not be consistently elevated in these patients, reducing their reliability as diagnostic tools [10]. This variability, coupled with the absence of established diagnostic criteria for extraintestinal *C. difficile *infections, underscores the importance of clinical vigilance and advanced imaging modalities in identifying these rare cases.

Due to the scarcity of cases, there is no standardized treatment protocol for *C. difficile* splenic abscesses [10-12]. Antibiotic therapy is often customized to the patient’s specific clinical presentation, comorbidities, and infection source [10]. Although intravenous vancomycin and metronidazole are commonly employed, their efficacy in managing splenic abscesses caused by *C. difficile* remains uncertain, and patients frequently require extended treatment durations [2,10,11]. Additionally, the emergence of antibiotic resistance in *C. difficile* isolates, particularly against metronidazole, adds another layer of complexity to treatment [10]. The potential for recurrence necessitates prolonged and closely monitored antibiotic regimens to mitigate the risk of a relapse.

Surgical intervention plays a pivotal role in managing splenic abscesses and is often critical to achieving source control. Percutaneous drainage is generally the preferred method, as it reduces bacterial load and enhances antibiotic penetration [1,2,11]. However, a splenectomy may be warranted in cases where drainage fails, the abscess is multilocular, or the patient’s condition remains unstable despite conservative measures. This case demonstrated the importance of interventional radiology in facilitating fluid drainage, which, combined with targeted antibiotic therapy, contributed to the patient’s clinical improvement.

While the exact mechanisms underlying the extraintestinal spread of* C. difficile* are not fully understood, the pathogen’s ability to form spores likely contributes to its persistence and recurrence in various anatomical sites [10]. Disruption of the intestinal barrier, often due to surgery, trauma, or underlying gastrointestinal conditions, is believed to play a major role in *C. difficile*’s ability to migrate beyond the colon [9-12]. This case highlights how gastrointestinal surgeries and barrier disruptions may predispose patients to rare manifestations of* C. difficile*, such as splenic abscesses, underlining the importance of careful monitoring in patients with extensive surgical histories.

This case contributes to the limited pool of knowledge on extraintestinal *C. difficile* infections and emphasizes the need for more comprehensive research. Large-scale studies or randomized trials are essential to improve the understanding of the epidemiology, pathogenesis, and optimal treatment strategies of* C. difficile* to help improve outcomes in these unusual presentations. Establishing standardized guidelines could enhance diagnostic accuracy, guide therapeutic decisions, and ultimately improve patient outcomes in cases of splenic abscesses and other extraintestinal manifestations of *C. difficile*.

## Conclusions

This case illustrates the complexities involved in diagnosing and managing an extraintestinal* Clostridioides difficile* infection presenting as a splenic abscess. Extraintestinal manifestations of *C. difficile*, especially splenic abscesses, are exceedingly rare and present unique challenges in terms of both clinical recognition and therapeutic decision-making. The absence of standardized diagnostic and treatment protocols, coupled with variable clinical presentations and potential antibiotic resistance, underscores the need for individualized care and close interdisciplinary collaboration.

Advanced imaging, timely intervention by interventional radiology, and carefully selected antimicrobial therapy are crucial in managing such rare infections. While surgical drainage played a pivotal role in this case, it also highlighted the potential need for prolonged antibiotic courses and vigilant follow-up to prevent recurrence. This case contributes to the growing, albeit limited, body of literature on extraintestinal *C. difficile* infections and reinforces the need for further research to guide diagnosis, optimize treatment, and improve outcomes for patients with similar complex presentations.
